# A nonparametric bootstrapping method for synthetically generating daily precipitation, water supply, and irrigation demand for rainwater harvesting system storage sizing

**DOI:** 10.1016/j.mex.2019.10.025

**Published:** 2019-11-12

**Authors:** Kurt Wurthmann

**Affiliations:** Nova Southeastern University, United States

**Keywords:** Nonparametric bootstrapping method for rainwater harvesting system storage sizing and reliability determination, Bootstrapped rainfall generation, Cistern sizing and reliability, Numerical mass balance analysis, Seasonality effects

## Abstract

This article describes a nonparametric bootstrapping method for synthetically generating daily precipitation, water supply, and irrigation demand for rainwater harvesting (RWH) system storage sizing and reliability determination. The method is illustrated using the case example of determining storage size and associated reliability outcomes for residential RWH systems that provide for the outdoor landscape irrigation demands of single-family homes in Broward and Palm Beach Counties, located in Southeast Florida, U.S.A. The method is useful not only for individual property owners, RWH system designers, and contractors, but also for policy makers who wish to analyze potential savings in water and energy amounts and costs that could result from widespread deployment of residential RWH systems, as discussed in Wurthmann (2019). The method can be easily implemented in Excel and is unique in its combination of:

• precision – determines daily levels of precipitation, water supply, and irrigation demand, incorporating the effects of seasonality,
• adaptability – user specified historical rainfall data and functional relationships between precipitation and water supply and demand are fully customizable, and
• portability – the nonparametric bootstrapping approach overcomes the key challenge posed by parametric stochastic methods; that statistical relationships describing rainfall processes derived in one location are likely not applicable to other locations

## Graphical abstract

**Figure:**
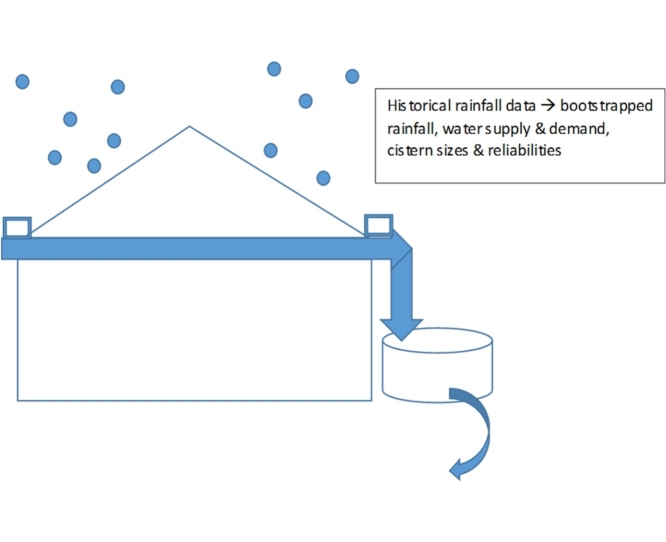

**Specification Table**

**Table:** 

Subject Area:	Environmental Science
More specific subject area:	Rainwater harvesting systems
Method name:	Nonparametric bootstrapping method for rainwater harvesting system storage sizing and reliability determination
Name and reference of original method:	Storage and Reliability Estimation Tool (SARET) [2]
Resource availability:	n/a

## Method details

This article describes a nonparametric bootstrapping method that was used to determine household-level rainwater harvesting (RWH) system storage size and reliability outcomes for subsequent use in an analysis of potential savings in water and energy amounts and costs from the widespread deployment of the system across Broward and Palm Beach Counties, located in Southeast Florida, U.S.A., in Wurthmann [1]. The method was implemented in Excel and designed to determine storage size and associated reliability outcomes for RWH systems that provide for the outdoor landscape irrigation demands of single-family detached homes in the regions of interest. Simple adjustments to the input functions relating daily rainfall to water supply and demand would enable the method to provide the same outcomes for RWH systems that provide for indoor or indoor and outdoor demands of different types (e.g. potable and/or non-potable demands), for homes or commercial or industrial complexes of varying types (e.g. farms, multi-family residences, businesses, etc.).

The method described in this paper was implemented using simple Excel spreadsheets to provide needed analyses and output, based on customizations of the descriptions of some of the key elements of the “Storage and Reliability Estimation Tool (SARET)” [2]. SARET is an ingenious and sophisticated tool that can be downloaded and used to provide a range of useful information focused on RWH system reliability, depending on user specified input. SARET is described as requiring 25 years of daily rainfall data as input (or “modification to work properly with a longer or shorter dataset”), user input on several pre-determined variables, and “several minutes to several hours to run, depending on the user’s selection.” [2] SARET offers many advantages, including providing a ready-to-use, sophisticated tool for RWH system reliability assessment. However, some applications may require customizations and be better served through the development of alternative tools. Creation of these alternative tools can be facilitated by considering some of the innovations and insights provided by the developers of the SARET tool.

The method described in the present paper was developed as an alternative to seeking to obtain and implement SARET using rainfall data that does not match the 25-year format required by this tool and then utilize SARET’s output, whose description suggests it might not be ideally suited to the needs of the subsequent analyses to be performed in Wurthmann [1]. In particular, the subsequent analyses called for in Wurthmann [1] require multiple series of numeric output values for storage size and associated reliability outcomes for residential RWH systems in multiple regions, based on bootstrapped mass balance analyses, considering synthetically generated daily rainfall amounts, derived from 11 years of rainfall data, and linked to daily household-level RWH system water supply and irrigation demand values.

The customized, bootstrapping method for RWH system storage sizing described in the present paper offers the following unique advantages over pre-existing methods: First, the method can be easily implemented as an Excel spreadsheet, providing full transparency concerning the operations applied to the input data. Second, the method can be easily adjusted to accommodate input consisting of a series of daily rainfall data, covering any time period. Third, the method allows users to precisely define daily irrigation demand as any function of daily precipitation, rather than only as a function of some discrete number of previous days without rainfall, as in SARET. Fourth, the method allows users to precisely define daily water supplied to the RWH system as any function of daily precipitation, rather than only as a function of a predetermined set of user input values related to catchment and first flush characteristics, as in SARET. Fifth, the method allows customization of synthetic rainfall generator parameters related to the effects of seasonality. Sixth, the method allows users to identify specific storage size and associated reliability values at any desired level of precision by simply increasing or decreasing the number of estimates of the RWH system storage required to supply the desired demand without failure during user specified time periods.

The remainder of this article is organized as follows. The next section describes the general mathematical model and algorithm for the numerical implementation of the sequent peak mass balance technique for storage sizing, which was employed in the method described in this paper. The subsequent two sections describe the derivation of the functional relationships between daily rainfall amounts and: (1) required daily outdoor irrigation demand (Section Functional relationship between daily rainfall and household water demand for irrigation); and (2) daily volumes of water captured in RWH system catchments and supplied to the system cisterns (Section Functional relationship between daily rainfall and water captured and supplied to household RWH system cisterns) in the two counties of interest (Broward and Palm Beach Counties). The following section describes the algorithm for incorporating the effects of seasonality into the nonparametric bootstrapping method for synthetically generating daily amounts of rainfall and related water supply and irrigation demand values. The penultimate section provides additional details concerning the method for generating and recording estimates of the RWH system storage required to supply desired demands without failure during each year of operation and how a series of these estimates can provide reliabilities associated with each storage size estimate. The final section provides some concluding remarks.

### General mathematical model and algorithm for the numerical implementation of the sequent peak mass balance technique for storage sizing

The general mathematical model and the algorithm for its numerical implementation for sizing the required storage for the RWH system, at the level of the individual household, represent mass balance sequent peak techniques [[3], [4], [5]]. Many years ago, water storage reservoirs were designed to be large enough to supply target amounts of water demand during the driest year in the record. However, Rippl [5] introduced the notion that several mild droughts in sequence will require larger storage than that required by the single driest year. Accordingly, inflows, outflows, and storage in reservoirs should be considered as a time series rather than a collection of independent outcomes, treated separately. Rippl’s method identifies “the smallest storage capacity … necessary to supply the desired [demand] …without failure throughout the whole period under consideration … This storage capacity is equal to the storage that would be depleted only in the most severe critical period” [3].

The process involves determining the required storage by integrating the residual mass curve (i.e. cumulative supply – demand), per the following equation:

$$Z_{t}=\int_{0}^{t}\left(x-q\right)d\tau =\int_{0}^{t}x\;d\tau -\int_{0}^{t}q\;d\tau =X_{t}-Q_{t}$$

Where X = supply; Q = Demand; and T = time

The algorithm for the numerical implementation of the mass balance approach used in the present research for finding the smallest storage capacity necessary to supply the desired demand without failure throughout the whole period, involved calculating the maximum cumulative deficit Kt on any day of the year, for 1000 bootstrapped sample years. The algorithm used to determine the maximum cumulative deficit Kt on any day of the year is as follows:

$$\begin{array}{l} K_{t}=\left\{\begin{array}{ll} \text{deman}{\text{d}}_{t}-\text{suppl}{\text{y}}_{t}+K_{t-1}; & \text{if}\;\text{positive}\\ 0; & \text{otherwise} \end{array}\right)\\ \text{Storage Required}-\max\left(K_{t}\right) \end{array}$$

The analysis for the case example described in the present paper, involved implementing the above algorithm in Excel using data on local historical daily rainfall amounts for a period of 11 years in Fort Lauderdale, Florida, and functional relationships, at the level of the individual household, between daily rainfall amounts and required daily outdoor irrigation demands and amounts of rainfall collected in RWH system catchments for supply to cisterns.

#### Functional relationship between daily rainfall and household water demand for irrigation

The functional relationship, at the level of the individual household, between daily rainfall amounts and required daily outdoor irrigation demand in Broward and Palm Beach Counties was determined as follows. First, the required application rate of water to the landscaped area of 0.5 in., once per week, year round [6,7], was converted to a constant demand amount of 0.00595 feet per day. Second, the difference between this constant daily requirement for irrigation and the amount of rain that fell each day was calculated, with the difference set equal to zero if it was a negative number (which happened on days when the amount of rainfall exceeded the irrigation needs). Third, this difference was multiplied by the outdoor landscaped area requiring irrigation to produce the volume of household water demand required on any day for landscape irrigation.

The outdoor landscaped area requiring irrigation was calculated using the following four steps: Step 1, census data was used to determine the percentages of single-family homes in the counties of interest, based on numbers of bedrooms [8]. Step 2, National Association of Home Builders’ (NAHB) data on the average square footage of homes for sizes corresponding to numbers of bedrooms [9] was multiplied by the census data to determine the weighted average square footages for detached, single-family homes of 2223.45 and 2284.80 in Broward and Palm Beach Counties, respectively. Step 3, average lot sizes for the two counties of interest were determined using Florida Department of Revenue Property Tax data files [10]. Step 4, outdoor landscaped area requiring irrigation was estimated as 50 % of the difference between average lot sizes and square footage of homes, or 3198.78 and 5233.60 square feet, for Broward and Palm Beach Counties, respectively [2].

The method described in the present paper allows the functional relationship between daily rainfall and household water demand for irrigation to be easily adjusted to accommodate the needs of the analyst. For example, adjustments to the application rate of water to the landscaped area and size of the area requiring irrigation would be needed for different regions, climates, vegetation types, and house and lot sizes. Functional relationships between daily rainfall and household water demand for other outdoor purposes (e.g. vehicle/building washing) or indoor non-potable (e.g. toilet flushing) or potable (e.g. drinking water) purposes could also be established and incorporated into the method described in the present paper.

#### Functional relationship between daily rainfall and water captured and supplied to household RWH system cisterns

The functional relationship, at the level of the individual household, between daily rainfall amounts and volumes of water captured in RWH system catchments and supplied to RWH system cisterns in Broward and Palm Beach Counties was determined as follows. First, the surface areas of the catchments in the two counties were set equal to the average home sizes of 2223.45 and 2284.80 square feet in Broward and Palm Beach Counties, respectively. This approach is consistent with the fact that the majority of homes in the two counties are ranch style homes. Second, daily volumes of water captured in RWH system catchments and supplied to RWH system cisterns in Broward and Palm Beach Counties were determined by simply multiplying daily rainfall amounts by corresponding catchment areas.

The method described in the present paper allows the functional relationship between daily rainfall and water captured and supplied to household RWH system cisterns to be easily adjusted to accommodate the needs of the analyst. For example, adjustments could be made to consider such factors as differing home sizes and types, first flush amounts and frequencies, or system evaporation losses.

### Algorithm for incorporating the effects of seasonality into the nonparametric bootstrapping method for synthetically generating daily amounts of rainfall and related water supply and irrigation demand

The synthetic rainfall generation algorithm used in the present study employed a database of historical daily precipitation amounts in Fort Lauderdale, Florida, for the 11 year period from 2007 to 2017 [11]. For each target day of the year, the rainfall amount generated (and related water supply and irrigation demand values) corresponded to the historical amount of rainfall on a randomly selected day, from a 30-day window centered on the target day (i.e. the target day, itself, the 15 days preceding it, and the 14 days following it), from a randomly selected year, between 2007 and 2017. The 30-day window wraps around at the beginning and end of the period, such that the 15 days preceding January 1, 2007 are December 17–31 of 2017 and the 14 days following December 31, 2017 are January 1–14 of 2007. For example, if the target day is December 30 and the randomly selected year is year 10 (2017) and the randomly selected day is day 17 (within the 30-day window centered on December 30), then the rainfall amount generated for that target day would be the historical amount of rainfall on January 1, 2007. If the target day is December 31 and the randomly selected year is year 10 (2017) and the randomly selected day is day 28 (within the 30-day window centered on December 31), then the rainfall amount generated for that target day would be the historical amount of rainfall on January 13, 2007.

Thus, the amount of rainfall generated for each target day of the year is randomly selected from a sample of 30 days × 11 years = 330 possible historical rainfall amounts. By randomly selecting rainfall amounts from within a moving, 30-day window centered on the target day, rather than simply randomly selecting rainfall amounts from any day within the 11-year period, the algorithm incorporates the effects of seasonality [2]. By randomly selecting rainfall amounts from within a moving, 30-day window centered on the target day, rather than simply randomly selecting rainfall amounts from just one of the 11 single days that exactly correspond to the target day, within the 11-year period, the algorithm draws from a sample that is more meaningful statistically (i.e. a sample of 330 possible rainfall amounts versus a sample of just 11 possible rainfall amounts) [2].

Note that the algorithm in the method described in the present paper uses random year 0 to indicate year 2007 and random year 10 to indicate year 2017 and uses random day zero to indicate the day 15 days preceding the target day and random day 29 to indicate the day 14 days following the target day. Users could easily adapt the algorithm for use with historical rainfall data from periods of time that are longer or shorter than the 11-year period used in the present method by simply adjusting the random year references. Further, users could easily adapt the algorithm to consider seasonality effects based on a longer or shorter period than the 30-day moving window used in the present method by simply adjusting the random day references.

### Additional details concerning the method for estimating RWH system storage size and reliability outcomes

The Excel implementation of the method described in the present article involved creating a database of historical daily precipitation amounts in Fort Lauderdale, Florida, for the 11-year period from 2007 to 2017 [11]. Within this database, year 0 corresponded to year 2007 and year 10 to year 2017 and day zero corresponded to the day 15 days preceding the target day and day 29 to the day 14 days following the target day. Target days are the days of the year for which rainfall amounts are to be synthetically generated. For each of the 365 target days in any given year of synthetically generated rainfall amounts, the Excel program identifies a matching index year and day within the database of 11 years of daily precipitation amounts using the functions INT(11*RAND()) and INT(30*RAND()), respectively. The rainfall amount on the matching index year and day within the database of 11 years of historical daily precipitation amounts is assigned to the target day. The functional relationships between daily rainfall and household water demand for irrigation and water captured and supplied to household RWH system cisterns described in the third and fourth sections of this paper, respectively, were used to transform synthetically generated rainfall amounts for each day of the year into synthetically generated daily water demand and supply.

For each of the 365 target days in any given year of synthetically generated rainfall amounts, the Excel program calculates a daily water deficit as the daily water demand minus the daily water supply. The Excel MAX function is used to calculate a running total of the cumulative water deficit for the entire year, on each day of the year, as the maximum of zero or the sum of the prior year’s cumulative deficit plus the current year’s deficit. The Excel MAX function is used a second time to calculate the maximum of the cumulative deficit amounts throughout the entire year of synthetically generated daily rainfall (and water demand and supply) amounts, which represents the smallest storage capacity necessary to supply the daily demand without failure throughout the entire year. Since the RAND function generates a new random number every time the Excel worksheet recalculates, with each recalculation the worksheet generates a new, randomly-generated “smallest storage capacity necessary to supply the daily demand without failure throughout the entire year.”

In the present case example, the bootstrapping approach was used to compute 1000 estimates of the RWH system storage required to supply the desired demand without failure during one year periods in Broward and Palm Beach Counties. The 1000 estimates of required storage volumes for the RHW systems in Broward and Palm Beach Counties were sorted in ascending order. Table 1 shows the 910^th^ through the 950^th^ largest storage sizes required to supply the desired demand without failure during one year periods in Broward and Palm Beach Counties. Since there are 1000 estimates, each increment represents an increase of 0.1 percentile points for system reliability.

**Table 1:** The 910^th^ through the 950^th^ largest storage sizes required to supply the desired demand without failure during one year periods in Broward and Palm Beach Counties based on the nonparametric bootstrapping model.

Ascending Rank	Storage needed Broward (gallons)	Storage needed Palm Beach (gallons)
910	7491.64	15,881.01
911	7494.11	15,907.61
912	7514.73	15,912.02
913	7532.23	15,925.34
914	7541.27	15,951.78
915	7565.44	15,980.23
916	7583.75	16,145.19
917	7602.99	16,227.21
918	7610.16	16,278.08
919	7625.83	16,281.69
920	7637.12	16,286.3
921	7643	16,303.09
922	7643.79	16,337.16
923	7705.79	16,346.57
924	7721.73	16,417.79
925	7733.61	16,443.85
926	7756.19	16,483.35
927	7758.5	16,493.61
928	7771.8	16,500.63
929	7780.14	16,588.39
930	7809.39	16,728.97
931	7813.22	16,733.55
932	7825.48	16,738.41
933	7848	16,753.36
934	7848.55	16,791.7
935	7867.53	16,820.03
936	7888.19	16,842.06
937	7927.3	16,851.66
938	7930.9	16,866.92
939	7957.58	16,896.65
940	7996.03	16,899.55
941	8002.46	16,903.07
942	8009.51	16,948.85
943	8020.31	16,996.97
944	8046.36	17,029.05
945	8066.99	17,051.02
946	8067.15	17,057.59
947	8108.67	17,086.29
948	8125.4	17,090.47
949	8216.78	17,109.57
950	8236.49	17,151.93

Thus, according to Table 1, a storage of size greater than 7996.03 gallons (e.g. one 8000 gallon cistern or four, linked, 2000 gallon cisterns) would provide for the outdoor landscape irrigation demands at single-family homes in Broward County Florida with 94 % reliability. Similarly, according to Table 1, a storage of size greater than 15,980.23 gallons (e.g. one 16,000 gallon cistern or eight, linked, 2000 gallon cisterns) would provide for the outdoor landscape irrigation demands at single-family homes in Palm Beach County Florida with 91.5 % reliability. It would be a simple matter for users to increase or decrease the precision of the storage sizing and reliability outcomes in the method presented in this paper by simply increasing or decreasing the numbers of estimates generated.

### Concluding remarks

This article has described a simple, highly-adaptable nonparametric bootstrapping method for synthetically generating daily precipitation, water supply, and irrigation demand for rainwater harvesting (RWH) system storage sizing and reliability determination. A key advantage of nonparametric versus parametric techniques is that they avoid consideration of extreme rainfall amounts that would exceed any maximum daily precipitation amount that actually occurred during the period of the historical data considered. Further, bootstrapped models overcome challenges posed by limitations in data availability and uncertainty about the behavior of outcomes over repeated sampling and the true shape of sampling distributions [2]. Accordingly, this article’s method for determining storage size and reliability outcomes for RWH systems is portable and applicable to studies in other regions of the world.

While several pre-existing methods and tools are available for implementing nonparametric bootstrapping approaches to synthetically generate rainfall amounts and determine RWH system reliabilities, these pre-existing methods and tools may not always be ideally suited to incorporating available data and customized input parameters or providing output in a form that is most useful for particular, tailored needs. This situation was illustrated in the present article using a case example in which the nature of available data on historical daily rainfall, the specifications of the relationships between this data and irrigation demands, and the need to obtain multiple series of numeric output values for storage size and associated reliability outcomes for residential RWH systems in multiple regions could not be easily accommodated using existing tools.

To overcome these challenges, the present paper specified an algorithm for numerically implementing the mass balance approach for finding the smallest storage capacity necessary to supply a desired demand without failure throughout a specified period. Techniques for specifying the functional relationships between daily rainfall amounts and required daily outdoor irrigation demands and volumes of water captured in RWH system catchments and supplied to RWH system cisterns were described and illustrated for the case of two counties in Southeast Florida. An easily implemented algorithm for incorporating the effects of seasonality into the nonparametric bootstrapping method for synthetically generating daily amounts of rainfall and related water supply and irrigation demand was also described and illustrated for use with a database of historical daily precipitation amounts in Fort Lauderdale, Florida, for the 11 year period from 2007 to 2017. Explanations were provided of the method of placing a series of estimates of the RWH system storage sizes required to supply desired demands without failure during one year periods in ascending order to produce a matched set of storage size and reliability values. This method was illustrated using the case example of identifying storage sizes and associated reliabilities for RWH systems designed to provide for the outdoor landscape irrigation demands at single-family detached homes in Broward and Palm Beach Counties in Florida. Wurthmann [1] presents the application of this data in a subsequent study, analyzing potential savings in water and energy amounts and costs that could result from the widespread deployment of residential RWH systems for outdoor landscape irrigation in Southeast Florida.

## Declaration of Competing Interest

The authors declare that they have no known competing financial interests or personal relationships that could have appeared to influence the work reported in this paper.
